# Potential Role of the Bovine Rumen Microbiome in Modulating Milk Composition and Feed Efficiency

**DOI:** 10.1371/journal.pone.0085423

**Published:** 2014-01-22

**Authors:** Elie Jami, Bryan A. White, Itzhak Mizrahi

**Affiliations:** 1 Department of Ruminant Science, Institute of Animal Sciences, Agricultural Research Organization, Volcani Center, Bet Dagan, Israel; 2 Department of Molecular Microbiology and Biotechnology, The George S. Wise Faculty of Life Science, Tel Aviv University, Ramat-Aviv, Israel; 3 Department of Animal Sciences, University of Illinois Urbana-Champaign, Urbana, Illinois, United States of America; 4 Institute for Genomic Biology, University of Illinois at Urbana-Champaign, Urbana, Illinois, United States of America; Charité, Campus Benjamin Franklin, Germany

## Abstract

Ruminants are completely dependent on their microbiota for feed digestion and consequently, their viability. It is therefore tempting to hypothesize a connection between the composition and abundance of resident rumen bacterial taxa and the physiological parameters of the host. Using a pyrosequencing approach, we characterized the rumen bacterial community composition in 15 dairy cows and their physiological parameters. We analyzed the degree of divergence between the different animals and found that some physiological parameters, such as milk yield and composition, are highly correlated with the abundance of various bacterial members of the rumen microbiome. One apparent finding was a strong correlation between the ratio of the phyla Firmicutes to Bacteroidetes and milk-fat yield. These findings paralleled human studies showing similar trends of increased adiposity with an increase in Bacteroidetes. This correlation remained evident at the genus level, where several genera showed correlations with the animals' physiological parameters. This suggests that the bacterial community has a role in shaping host physiological parameters. A deeper understanding of this process may allow us to modulate the rumen microbiome for better agricultural yield through bacterial community design.

## Introduction

In recent years, the microorganisms residing in the gut of multicellular organisms, termed gut microbiome, have been shown to play an important role in their host's physiology [1], [2]. In mice and humans, a link has been demonstrated between the gut microbiota and the physiological features of energy-harvesting abilities, with genetically predisposed obese mice exhibiting a different ratio of the phyla Firmicutes to Bacteroidetes [3], [4]. The transfer of microbiota from obese mice to lean mice resulted in significant physiological changes in the latter, related to tissue adiposity, suggesting a causative effect of the microbiota on its host's physiology. The conclusion was that the “obese” microbiome has an increased capacity to harvest energy from the diet [4]. Recently, gnotobiotic mice colonized with obese human microbiota exhibited increased adiposity, but when these same mice were exposed to lean human microbiota transplanted in the same manner, the obese microbiota was invaded by bacterial components of the lean microbiota, causing a decrease in the obese phenotype [5].

The bovine rumen houses a complex microbiota that is responsible for cattle's ability to convert indigestible plant mass into energy [6]. This ability is of tremendous importance for mankind, as domesticated animals are a crucial intermediate between light energy harvested via photosynthesis and the production of digestible compounds such as milk and meat [7], [8]. The rumen functions as a pregastric anaerobic fermentation chamber inhabited by a highly dense microbial community composed of microorganisms from all domains of life, 95% of which are bacteria [9]. Fermentation products of rumen microbial activity—mainly volatile fatty acids—serve as a major source of energy for the animal [7], [8]. These fermentation products have a direct effect on the animal's physiological parameters, such as milk composition [10]. Using denaturing-gradient gel electrophoresis (DGGE), a connection between volatile fatty acid composition and rumen bacteria was suggested, by examining microbial differences between efficient and inefficient cows [11]. High inter-animal variation exists in the stability of the rumen microbiome [9], [12], but intra-animal variation is quite low [13]. Therefore, it is tempting to speculate that these inter-microbiome variations might be linked to the physiological parameters of their individual hosts. In the present study, by analyzing the whole rumen bacterial communities of 15 dairy cows—previously sequenced in our lab, and comparing them to the cows' production parameters and milk composition, we explored the possible link between bacterial components of the rumen microbiome and the physiological parameters of the animal host during lactation.

## Materials and Methods

### Animal handling and sampling

The experimental procedures used in this study were approved by the Faculty Animal Policy and Welfare Committee of the Agricultural Research Organization (ARO), approval number IL-168/08, Volcani Research Center, and were in accordance with the guidelines of the Israel Council on Animal Care.

Healthy 2-year-old Israeli Holstein Friesian lactating cows were housed together (n = 15) at the ARO dairy farm in Bet Dagan, Israel. The cows were selected for similar physical condition—age and weight—and were sampled during their first pregnancy, at the same stage of lactation. The cows were fed a diet consisting of 30% roughage and 70% concentrate (Table S1) ad libitum, provided once a day, which is the standard practice and feeding regimen in our facilities. Ruminal contents, collected via the cow's mouth using a stainless-steel stomach tube with a rumen vacuum sampler, were taken 1 hour after the morning feeding. Samples were immediately transferred to CO_2_-containing centrifuge bottles to maintain anaerobic conditions, and kept on ice. Within 1 h of collection, the ruminal samples were processed in the laboratory.

### Cow physiological parameters

Physiological parameters were recorded using an in-house automated–computerized monitoring system designed to identify individual cows electronically and automatically record each cow's parameters [14]. Milk yield (kg) of each cow was recorded for each milking and a daily average was calculated by automatic meters (Afimilk SAE, Afikim, Israel). Milk samples were collected in three sequential milkings on a weekly basis from the day cows were introduced to the high-concentrate diet until rumen sampling 5 weeks later. Analysis of fat, true protein, and lactose in the milk was performed by infrared analysis (Israeli Cattle Breeders Association laboratory, Caesaria, Israel) using a Milkoscan 4000 (Foss Electric, Hillerod, Denmark). Both residual feed intake (RFI) and feed-conversion ratio (FCR) were calculated according to the National Research Council [15]. RFI evaluates energetic efficiency according to the difference between the animal's actual feed intake and its estimated feed intake over a specified period of time [15]–[17]. Animals with low RFI values are considered to be more energetically efficient than those with high values. The independence of this method from growth and body size makes it suitable for comparisons between animals.

### Bacterial extraction and DNA purification

Bacterial isolation was performed as described previously [12]. Briefly, samples were homogenized for 2 minutes in a blender, which was washed with 70% ethanol and distilled water between samples to avoid cross-contamination, and then centrifuged at 10,000 *g*. The supernatant was discarded and the pellet was dissolved 1∶4 (g∶ml) in extraction buffer (100 mM Tris-HCl, 10 mM ethylenediaminetetraacetic acid [EDTA], 0.15 M NaCl pH 8.0). The samples were then incubated at 4°C for 1 hour to maximize the release of particle-associated bacteria from the ruminal contents [18]. This was followed by 15 minutes centrifugation at 500 *g* to discard plant particles while the bacterial cells remained in suspension [19]. The supernatant was then passed through four layers of new, sterile cheesecloth, and centrifuged (10,000 *g*, 25 minutes, 4°C), and the pellets were kept at −20°C until DNA extraction.

DNA extraction was performed as described by Stevenson and Weimer [18]. Briefly, cells were lysed by bead disruption with phenol, and phenol/chloroform extraction of DNA was performed. DNA was then precipitated using isopropanol and the precipitate was resuspended in Tris-EDTA buffer and stored at −20°C until analysis. Protocols for bacterial extraction and DNA purification were verified for reproducibility by performing duplicates for each sample and assessed by automated ribosomal spacer analysis (ARISA) for the whole bacterial community. Analysis of similarities (ANOSIM) was used in order to test whether there is a significant difference between the bacterial extraction and DNA purification methods coming from a given sample. This analysis revealed that there is no statistical difference between the replicates, indicating that the microbes and DNA extraction do not cause any differential bias across the samples. This can also be visualized by using cluster analysis dendrogram (Figure S3).

### 454 Tag amplicon pyrosequencing and data analyses

454 Amplicon pyrosequencing of the ruminal DNA samples was performed as described previously [12]. The sequencing was done at the Research and Testing Laboratory (Lubbock, TX) using primers covering the 103- to 530-bp region of the 16S rRNA gene sequence which corresponds to the V2 and V3 regions (107 F: 5′-GGCGVACGGGTGAGTAA-3′ and 530 R: 5′-CCGCNGCNGCTGGCAC-3′). The tagging and sequencing protocol was as described by Dowd *et al.* [20]. Data quality control and analyses were mostly performed using the QIIME pipeline [21]. The UCLUST method [22] was selected for operational taxonomic unit (OTU) clustering with degree of similarity between sequences defined as ≥97% and ≥94% for OTU identity at the species and genus level, respectively. We used the representative sequence of each OTU to remove chimeric sequences using the ChimeraSlayer algorithm [23]. OTUs which clustered only one or two reads were manually removed. After constructing an OTU table, taxonomy was assigned using the BLAST algorithm with the Greengenes 16S rRNA reference database found at http://blog.qiime.org designated “most recent Greengenes OTUs”. All sequences used for this study were publicly deposited in the MG-RAST server, I.D no. 4483775.3.

### Statistical analysis

Pearson correlation was used to correlate physiological parameters and bacterial composition using PAleontological STatistics (PAST) software [24] and plotted using the corrplot R package [25].

## Results and Discussion

**Figure 1:**
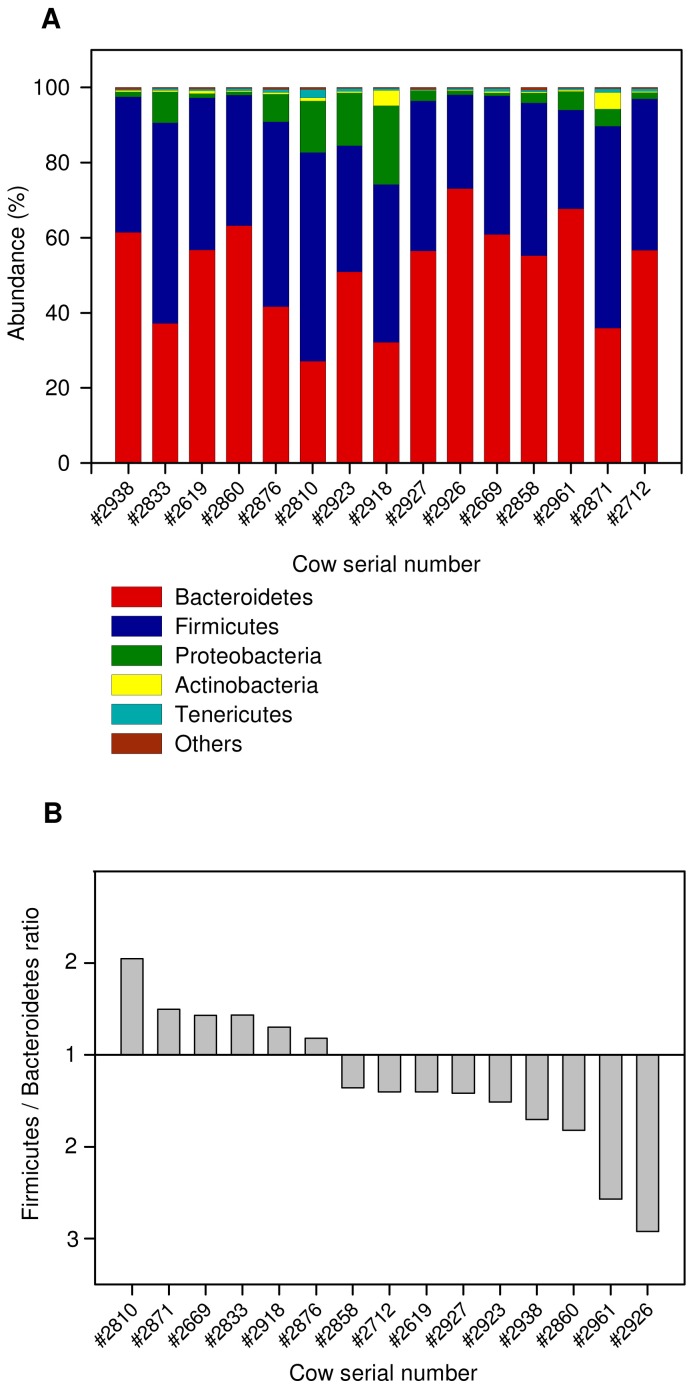
Phylum level composition. (A) Stacked bar plot showing the phylum-level composition for each individual cow rumen sampled. (B) Ratio of Firmicutes to Bacteroidetes.

**Figure 2:**
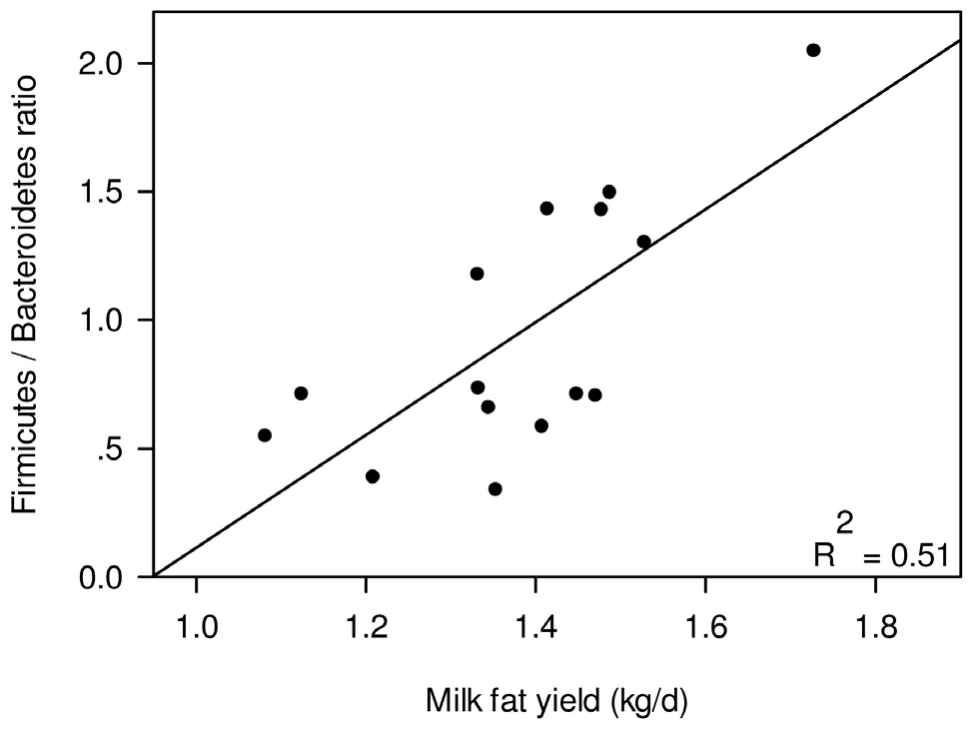
Correlation between milk-fat yield and Firmicutes-to-Bacteroidetes ratio. Scatter plot showing the amount of fat produced per day for each cow (X-axis), vs. the Firmicutes-to-Bacteroidetes ratio. Each point represents one individual cow. R^2^ of the linear regression is shown in the upper right corner of the plot.

Our aim in this study was to determine whether there are any correlations between the bacterial community residing in the cow rumen and the physiology of the individual cow hosts. We analyzed 15 lactating dairy cows, whose ruminal bacterial communities had been previously pyrosequenced [12], under a high-energy diet (Table S1). Their rumen fluid was sampled during lactation and their physiological parameters were recorded and calculated. These included milk yield, milk content (carbohydrate, protein, and fat), pH, dry matter intake (DMI) and RFI, which serves to evaluate the animal's feed efficiency (Table S2). After quality-filtering based on length (<200 bp) and quality of the reads, we obtained 141,344 reads averaging 338 bp each (Table S3). Overall, 17 phyla were detected, but only 7 were found in all cows (Figure 1A). The three dominant phyla observed, in agreement with all studies of mammalian gut microbiota, were Bacteroidetes, Firmicutes and Proteobacteria, as previously described and reported in other mammalian gut studies [2], [12], [26]. However, there was a large variation in the abundance of the two main phyla—Bacteroidetes and Firmicutes—between the different animals [13]. Although Bacteroidetes was more abundant in most of the samples, some exhibited a higher percentage of Firmicutes compensating for a lower abundance of Bacteroidetes (Figure 1B). The ratio of Firmicutes to Bacteroidetes has been shown to affect energy harvesting and body fat in humans and mice [3], [4]. We therefore examined whether cattle physiological parameters correlate with a change in this ratio. The Firmicutes-to-Bacteroidetes ratio was found to be strongly correlated with daily milk-fat yield (Pearson R = 0.72, *P* = 2×10^−3^) (Figure 2). This finding mirrors that in mice, where a decreased amount of Bacteroidetes in the microbiota was correlated with increased fat in the blood and tissue [4].

**Figure 3:**
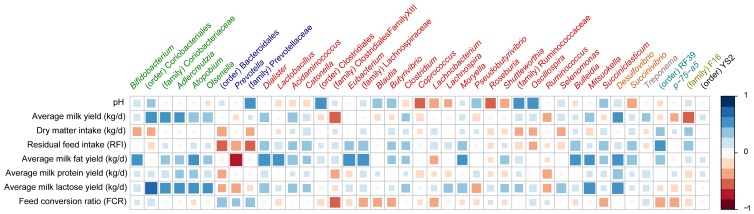
Correlation between efficiency parameter and genus abundance. Pearson linear correlation matrix of the dominant bacterial genera across the rumen samples. The genera were included in the matrix if they were in at least 50% of the cows and represented at least 0.1% of the bacterial community in at least one of the cows. Strong correlations are indicated by large squares, weak correlations by small squares. The scale colors denote whether the correlation is positive (closer to 1, blue squares) or negative (closer to −1, red squares) between the genera and the efficiency parameters. Color coding represents the phylum to which each genus belongs, as follows: Actinobacteria (green), Bacteroidetes (blue), Firmicutes (red), Proteobacteria (orange), Spirochaetes (purple), Tenericutes (light blue), TM7 (olive), Cyanobacteria (black).

**Figure 4:**
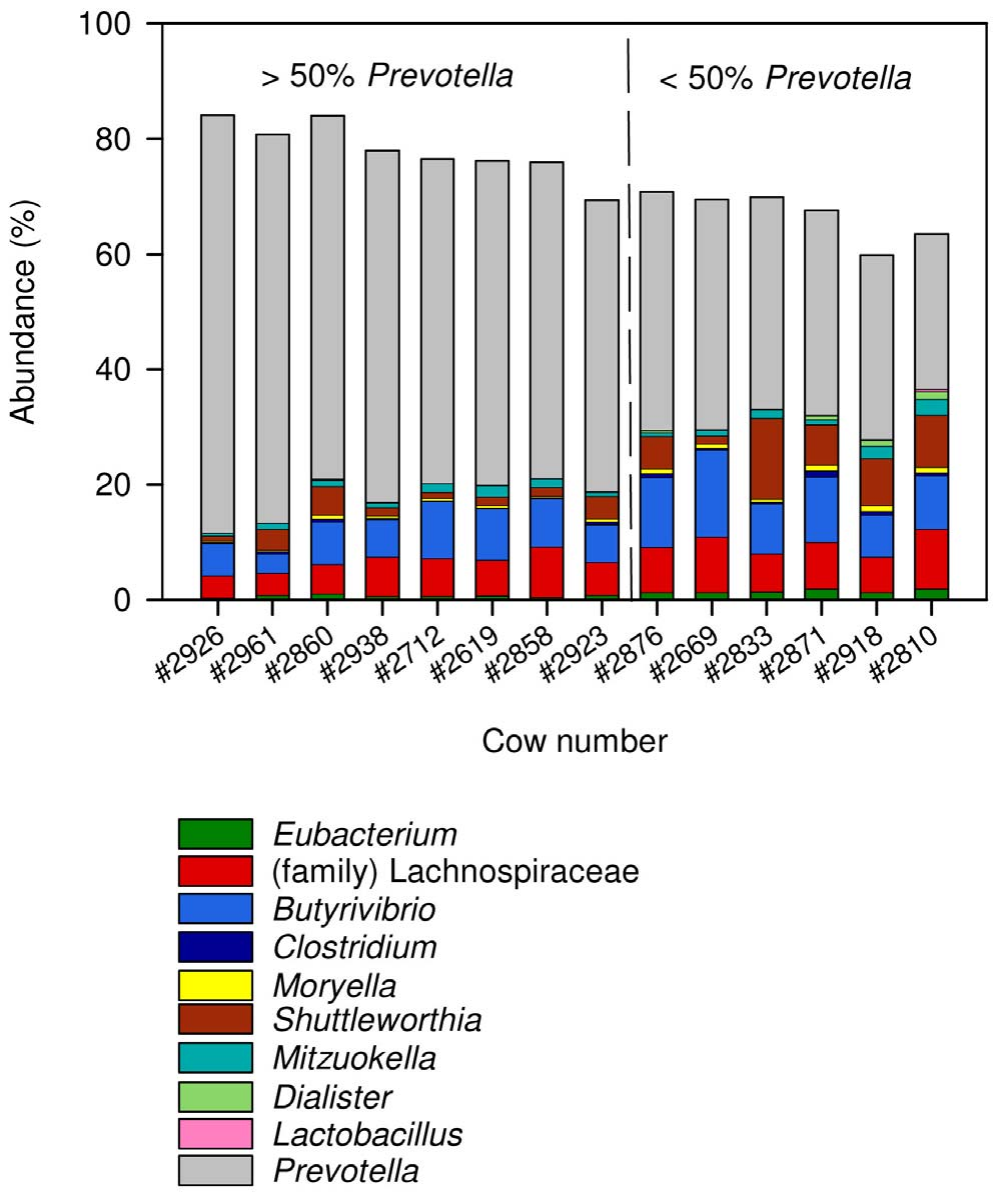
Abundance of genera within the phylum Firmicutes compared to the genus *Prevotella*. Stack bar showing the abundance of genera belonging to the phylum Firmicutes that were negatively correlated with *Prevotella* abundance. These included all genera that were in at least half of the cows sampled and constituted 0.1% of the reads in at least one cow. The gray portion of the bars represents the abundance of *Prevotella* (phylum Bacteroidetes). The dashed line separates samples with more than 50% *Prevotella* (left side) and from those with less than 50% *Prevotella*.

We then compared the physiological parameters with the microbiota at the genus level. To confirm adequate sequencing depth for these analyses, we generated rarefaction curves for each sample as a function of the number of observed OTUs (OTU≥94%, defined as genus level) and found our coverage to be sufficient for further analyses at the genus level (Figure S1). Overall, 151 genera were detected in our samples. We focused our analysis on the more abundant taxa, and only genera that were in at least half of the samples at over 0.1% of the microbiota in at least one animal were included in the analysis. Therefore, only 42 genera were compared to the physiological parameters. These included those found to be part of the core community in a previous study, i.e., shared by all of the animals sampled, and accounting for over 90% of the overall rumen bacterial OTUs [12]. Our assumption was that these 42 genera represent important components of the healthy rumen ecosystem, and would therefore be more likely to reveal a connection between host physiology and the bacterial community residing in its rumen. A correlation matrix was created to evaluate each of these genera with each physiological parameter (Figure 3). *Prevotella*, the most abundant genus in the samples (up to 72% in some samples), showed a significantly negative correlation (Pearson R = −0.69, *P* = 5×10^−3^) with milk-fat yield, explaining most of the Bacteriodetes' negative correlation to this parameter, as well as its correlation with Firmicutes-to-Bacteroidetes ratio. Firmicutes, on the other hand, was composed of many lower-abundance genera, only a fraction of which compensated for the decreasing abundance of *Prevotella* in the samples. One species of *Prevotella*, *P. bryantii*, has been associated with probiotic activities: cows inoculated with *P. bryantii* strain 25A had decreased lactate production [27]. That same study also showed an increase in milk fat during the weeks following inoculation. This effect, however, was caused by the inoculation of that one specific strain and did not reflect a general modulation by the genus *Prevotella*. Analysis of the genera from the phylum Firmicutes revealed that 9 out of the 23 genera analyzed (Figures 4 and S2) were more abundant in samples with low levels of *Prevotella*, and 5 of these were correlated with milk-fat yield; most of these belonged to the order Clostridiales—the genus *Eubacterium* (Pearson R = 0.62, *P* = 0.012) and the family Lachnospiraceae (Pearson R = 0.62, *P* = 0.014) (Figure 3), and some belonged to the class Negativicutes, only recently defined as such, and formerly members of the Clostridia, such as the genus *Dialister* (Pearson R = 0.64, *P* = 0.009). Some of the genera belonging to Firmicutes were of relatively similar abundance between the samples regardless of the abundance of *Prevotella*, whereas others were found in higher abundance in samples with a low abundance of *Prevotella*. Two genera, *Dialister* and *Lactobacillus*, were almost nonexistent in samples with over 50% *Prevotella*, whereas they were present in all samples with less than 50% *Prevotella* (Figures 4 and S2). Genera belonging to other phyla also showed a correlation with milk-fat yield, such as the genus *Desulfovibrio*, belonging to the Proteobacteria. From the phylum Actinobacteria, both *Bifidobacterium* and *Lactobacillus*, widely used as probiotics, also showed a positive correlation to milk-fat yield, along with the genus *Bulleidia*, belonging to the Firmicutes. Whereas the correlations between the microbiota and milk-fat yield were found to be the strongest, we also detected both negative and positive correlations with other parameters related to the host's physiology and milk composition, including milk lactose and protein contents. With respect to host physiology, some bacteria were correlated with the variation in ruminal pH between cows, such as the genus *Rosburia* (Pearson R = −0.5, *P* = 0.06). One study reported that members of this taxon are affected by changes in pH [28], with optimal growth under slightly acidic conditions. Those authors suggested that this genus is affected by either pH or competitors that emerge at more neutral pH values. Interestingly, significant positive correlations were observed between four genera, all belonging to the order Coriobacteriales, and milk-lactose content. These were *Atopobium* and *Adlercreutzia*, and two unknown genera belonging to the order Coriobacteriales, one of them also positively correlating with average milk yield (Pearson R = 0.57 *P* = 0.027) (Figure 3). The fact that these taxa are phylogenetically related suggests that they share functions that affect the host's physiology in a similar manner. In addition, both *Mitsuokella* and *Desulfovibrio* were positively correlated with milk-lactose yield (Pearson R = 0.59 for both genera). No significant correlation was detected between the bacterial community and RFI; however, a positive, albeit nonsignificant correlation (Pearson R = 0.51, *P* = 0.055) was detected between an unclassified genus from the putative order RF39, found in all of the animals sampled, and RFI. This genus, although little studied, is found in many gut environments [12], [29], including the rumen, hinting at a potentially crucial role in the gut of many species. Nevertheless, additional sampling is required to determine whether this taxon is associated with feed efficiency in cattle.

Overall, our findings in this study show remarkable similarities with those in other mammalian host systems regarding their interaction with the gut microbiome. This, along with our previous study examining rumen colonization—which also revealed mechanistic similarities between different organisms and their digestive strategies [30]—suggests an underlying mechanism of acquisition and energy utilization that may be common to many of the studied gut systems, regardless of the apparent phylogenetic distances between the hosts.

Our current study suggests a connection between the physiological parameters of dairy cattle and their resident rumen bacteria and reveals potential candidate taxa that may prove useful for future inoculation studies. Additional work is needed to evaluate the causative relationships between the host and gut microbiota in cattle.

## Supporting Information

Figure S1 **Genus-level rarefaction curves of rumen microbiota.** Rumen microbiota from each of the 15 individual animals were sampled according to their 16S rRNA gene sequences.
(TIF)

Figure S2 **Abundance of genera of the phylum Firmicutes compared to the genus** ***Prevotella*** **.** Stack plot showing the abundance levels of each of the 23 genera belonging to the phylum Firmicutes included in the correlation analyses. These include all genera that were in at least half of the cows sampled and constituted 0.1% of the reads in at least one cow. The gray portion of the bars represents the abundance of *Prevotella* (phylum Bacteroidetes).
(TIF)

Figure S3 **Assessment of the robustness of bacterial extraction and DNA purification protocols used in this study.** Dendrogram showing the degree of Bray–Curtis similarity between each sample and the technical duplicates for the bacterial extraction and purification protocols. Each animal sampled is represented by a different color. Samples with the same serial designation are the technical PCR duplicates and the ones with the letter “b” added to the same serial number represent the duplicates for the bacterial extraction and purification protocols.
(TIF)

Table S1 Formulated ingredients in g/kg dry matter (DM) of the basic total mixed rations given to lactating dairy cows.
(DOCX)

Table S2 Production parameter values and SEM for each individual cow.
(DOCX)

Table S3 Length and number of reads per animal sampled after quality filtering and removal of chimeric sequences and singletons-doubletons.
(DOCX)
