# Supplementary figures and images for: Potential Role of the Bovine Rumen Microbiome in Modulating Milk Composition and Feed Efficiency
Source: PLoS One. 2014 Jan 22;9(1):e85423. doi: 10.1371/journal.pone.0085423 (PMC3899005; doi:10.1371/journal.pone.0085423)

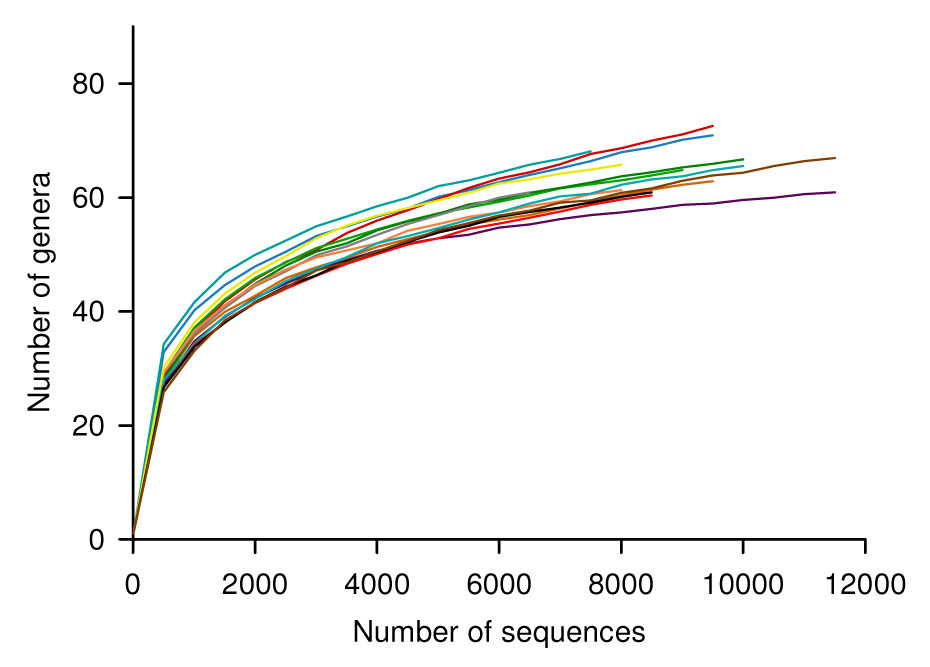

Supplement: Figure S1 — Genus-level rarefaction curves of rumen microbiota. Rumen microbiota from each of the 15 individual animals were sampled according to their 16S rRNA gene sequences. (TIF) [file pone.0085423.s001.tif]

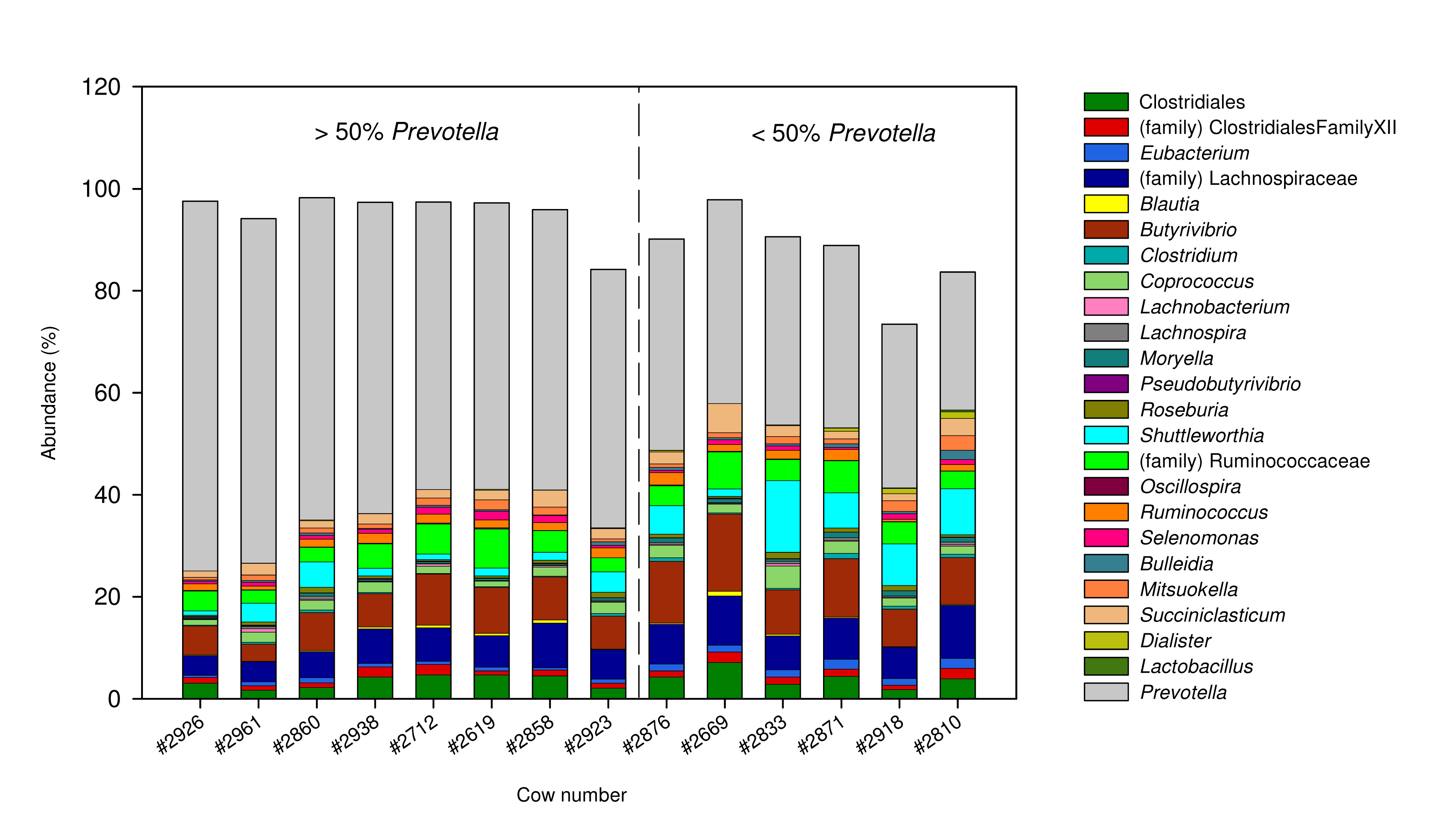

Supplement: Figure S2 — Abundance of genera of the phylum Firmicutes compared to the genus Prevotella . Stack plot showing the abundance levels of each of the 23 genera belonging to the phylum Firmicutes included in the correlation analyses. These include all genera that were in at least half of the cows sampled and constituted 0.1% of the reads in at least one cow. The gray portion of the bars represents the abundance of Prevotella (phylum Bacteroidetes). (TIF) [file pone.0085423.s002.tif]

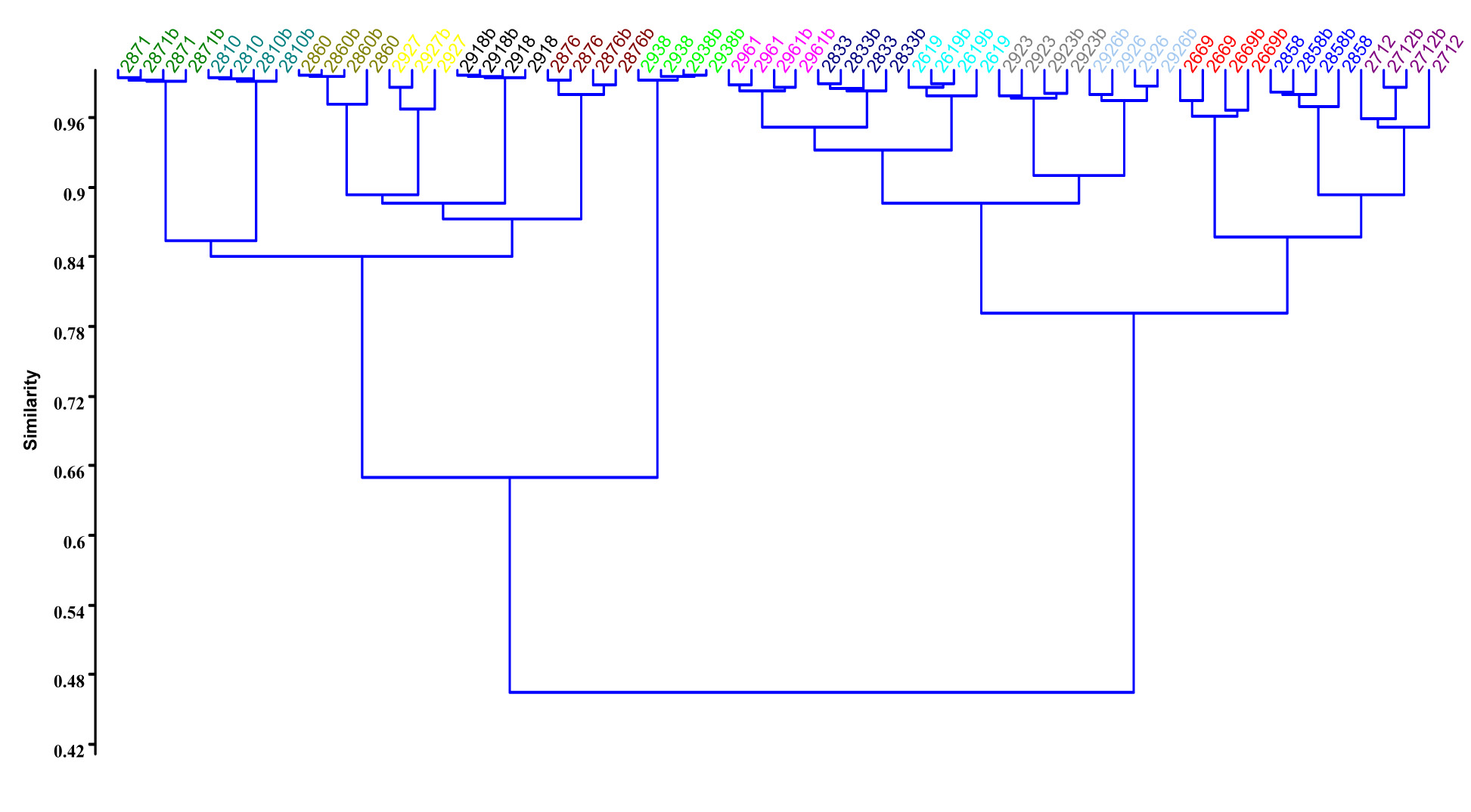

Supplement: Figure S3 — Assessment of the robustness of bacterial extraction and DNA purification protocols used in this study. Dendrogram showing the degree of Bray–Curtis similarity between each sample and the technical duplicates for the bacterial extraction and purification protocols. Each animal sampled is represented by a different color. Samples with the same serial designation are the technical PCR duplicates and the ones with the letter “b” added to the same serial number represent the duplicates for the bacterial extraction and purification protocols. (TIF) [file pone.0085423.s003.tif]
