# Supplementary material for: Potential Role of the Bovine Rumen Microbiome in Modulating Milk Composition and Feed Efficiency
Source: PLoS One. 2014 Jan 22;9(1):e85423. doi: 10.1371/journal.pone.0085423 (PMC3899005; doi:10.1371/journal.pone.0085423)
Supplement: Table S1 — Formulated ingredients in g/kg dry matter (DM) of the basic total mixed rations given to lactating dairy cows. (DOCX) [file pone.0085423.s004.docx]

**Table S1. Formulated ingredients in g/kg dry matter (DM) of the basic**

**total mixed rations given to lactating dairy cows.**

| Diet composition | |
| --- | --- |
| Wheat silage | 100 |
| Oat hay | 80 |
| Corn silage | 100 |
| Clover hay | 23 |
| Soybean hulls | 78 |
| Soybean meal (solvent-extracted) | 22 |
| Ground corn grain | 129 |
| Ground barley grain | 87 |
| Ground wheat grain | 44 |
| Whole cotton seeds | 20 |
| Corn gluten feed | 96 |
| Corn distillers dry grain | 89 |
| Rapeseed meal | 38 |
| Whey solids | 36 |
| NaHCO_3_ | 7.4 |
| NaCl | 6 |
| CaCO_3_ | 9 |
| Ca-LCFA^**^ | 14 |
| Soy Molasses | 17 |
| Urea | 4 |
| Trace mineral + vitamin mixture^*^ | 0.6 |

^*^ The trace mineral + vitamin mix contained (g /kg DM-1): Zn, 24; Fe, 24; Cu,

12.8; Mn, 24; I, 1.44; Co, 0.32; Se, 0.32; Vit. A, 16,000,000 IU; Vit. D3,3,200,000 IU;

Vitamin E, 48,000 IU.

^**^ Calcium salts of long-chain fatty acids.
