# Supplementary material for: Potential Role of the Bovine Rumen Microbiome in Modulating Milk Composition and Feed Efficiency
Source: PLoS One. 2014 Jan 22;9(1):e85423. doi: 10.1371/journal.pone.0085423 (PMC3899005; doi:10.1371/journal.pone.0085423)
Supplement: Table S2 — Production parameter values and SEM for each individual cow. (DOCX) [file pone.0085423.s005.docx]

**Table S2.** **Production parameter values and SEM for each individual cow.**

| Cow serial number | 2938 | 2833 | 2619 | 2860 | 2876 | 2810 | 2923 | 2918 | 2927 | 2926 | 2669 | 2858 | 2961 | 2871 | 2712 | average | SEM | %RSD^****^ |
| --- | --- | --- | --- | --- | --- | --- | --- | --- | --- | --- | --- | --- | --- | --- | --- | --- | --- | --- |
| pH^*^ | 6.72 | 6.17 | 7.09 | 6.66 | 6.85 | 6.54 | 5.84 | 6.54 | 6.39 | 6.84 | 7.15 | 6.4 | 6.3 | 6.39 | 6.32 | 6.55 | 0.09 | 5.32 |
| Average milk yield (kg/d)^**^ | 38.92± 0.43 | 41.69± 0.97 | 54.6± 0.54 | 43.87± 0.4 | 40.59± 0.37 | 46.41± 2.01 | 43.52± 0.32 | 48.52± 0.59 | 45.65± 0.44 | 42.8± 0.51 | 41.73± 0.38 | 45.58± 0.62 | 47.75± 0.37 | 50.19± 0.51 | 37.95± 0.37 | 44.65 | 1.15 | 9.9 |
| Dry matter intake (kg/d) | 25.72± 0.43 | 26.94± 0.27 | 23.6± 0.52 | 28.27± 0.52 | 33.3± 0.46 | 30.1± 0.51 | 32.42± 0.42 | 30.11± 0.52 | 30.42± 0.21 | 31.18± 0.44 | 31.58± 0.36 | 32.27± 0.57 | 30.81± 0.35 | 29.95± 0.48 | 33.06± 0.44 | 29.16 | 0.72 | 8.8 |
| Residual feed intake (RFI)^***^ | -4.26 | 2.12- | 4.85- | 2.52- | 2.98 | 3.23 | 0.76 | 0.94 | 0.18- | 0.45- | 1.85 | 1.79 | 1.06 | 0.62 | 1.16 | 1.01 | 0.53 | 203 |
| Average milk fat yield (kg/d) ^**^ | 1.41± 0.014 | 1.41± 0.017 | 1.45± 0.019 | 1.08± 0.016 | 1.33± 0.022 | 1.73± 0.065 | 1.34± 0.015 | 1.53± 0.034 | 1.47± 0.014 | 1.35± 0.019 | 1.48± 0.01 | 1.33± 0.024 | 1.21± 0.033 | 1.49± 0.019 | 1.12± 0.009 | 1.37 | 0.04 | 10.9 |
| Average milk protein yield (kg/d) ^**^ | 1.31± 0.012 | 1.24± 0.025 | 1.43± 0.015 | 1.36± 0.014 | 1.25± 0.015 | 1.41± 0.06 | 1.35± 0.01 | 1.47± 0.016 | 1.55± 0.013 | 1.32± 0.014 | 1.43± 0.016 | 1.45± 0.022 | 1.42± 0.018 | 1.4± 0.013 | 1.2± 0.009 | 1.36 | 0.02 | 7.1 |
| Average milk lactose yield (kg/d) ^**^ | 1.87± 0.02 | 2.03± 0.05 | 2.64± 0.03 | 2.17± 0.02 | 2.02± 0.01 | 2.22± 0.11 | 1.35± 0.016 | 2.43± 0.031 | 1.57± 0.021 | 1.24± 0.025 | 2.04± 0.019 | 2.13± 0.028 | 2.34± 0.017 | 2.45± 0.025 | 1.8± 0.018 | 2.18 | 0.09 | 10.5 |
| Feed conversion ratio (FCR)^**^ | 0.63 | 0.52 | 0.69 | 0.57 | 0.62 | 0.52 | 0.55 | 0.55 | 0.53 | 0.56 | 0.47 | 0.52 | 0.58 | 0.53 | 0.45 | 0.55 | 0.02 | 11.3 |

^*^ pH during sampling.

^**^  Milk yield and composition values represent the average obtained from measurement of 3 consecutive weekly milkings for each cow for 5 weeks prior to rumen sampling.

^***^ Both residual feed intake (RFI) and feed conversion ratio were calculated once at the end of the milking measurments according to National Research Council (NRC, 2001).

^****^ %RSD was calculated as follows; (standard deviation / average) x100.
