# Supplementary material for: Potential Role of the Bovine Rumen Microbiome in Modulating Milk Composition and Feed Efficiency
Source: PLoS One. 2014 Jan 22;9(1):e85423. doi: 10.1371/journal.pone.0085423 (PMC3899005; doi:10.1371/journal.pone.0085423)
Supplement: Table S3 — Length and number of reads per animal sampled after quality filtering and removal of chimeric sequences and singletons-doubletons. (DOCX) [file pone.0085423.s006.docx]

**Table S3. Length and number of the reads per animal sampled after quality filtering,**  **chimeric sequences and singletons-doubletons removal.**

| Cow # | Mean read length | Number of reads per animal |
| --- | --- | --- |
| 2918 | 332 ± 72 | 9940 |
| 2669 | 348 ± 83 | 9690 |
| 2858 | 342 ± 81 | 9125 |
| 2961 | 334 ± 85 | 11532 |
| 2871 | 342 ± 72 | 7868 |
| 2712 | 339 ± 82 | 9536 |
| 2938 | 335 ± 81 | 10165 |
| 2833 | 342 ± 74 | 6858 |
| 2619 | 339 ± 81 | 8367 |
| 2860 | 336 ± 83 | 10241 |
| 2876 | 338 ± 77 | 8135 |
| 2810 | 341 ± 73 | 7322 |
| 2923 | 331 ± 71 | 8803 |
| 2927 | 336 ± 81 | 8537 |
| 2926 | 334 ± 86 | 15225 |
| Average | 338 ± 80 | 9422 ± 2020 |
